# Equinatoxin II: How a Cationic Pore-Forming Sea Anemone Toxin Drives Nodal Swelling of Myelinated Nerve Fibers

**DOI:** 10.3390/md24050187

**Published:** 2026-05-21

**Authors:** Evelyne Benoit, Robert Frangež, Gilles Ouanounou, Frédéric A. Meunier, Dusan Šuput, Jordi Molgó

**Affiliations:** 1 Service d’Ingénierie Moléculaire Pour la Santé (SIMoS), EMR CNRS 9004, Département Médicaments et Technologies Pour la Santé (DMTS), Institut des Sciences du Vivant Frédéric Joliot, Commissariat à l’énergie atomique et aux énergies alternatives (CEA), Université Paris-Saclay, F-91191 Gif-sur-Yvette, France; evelyne.jbenoit@gmail.com; 2 Institute of Preclinical Sciences, Veterinary Faculty, University of Ljubljana, Gerbičeva 60, S-1000 Ljubljana, Slovenia; robert.frangez@vf.uni-lj.si; 3 UMR CNRS 9197, Institut des Neurosciences Paris-Saclay (NeuroPSI), Université Paris-Saclay, F-91400 Saclay, France; gilles.ouanounou@cnrs.fr; 4 Clem Jones Centre for Ageing Dementia Research, Queensland Brain Institute, School of Biomedical Sciences, The University of Queensland, St Lucia, QLD 4072, Australia; f.meunier@uq.edu.au; 5 Institute of Pathophysiology, Faculty of Medicine, University of Ljubljana, S-1000 Ljubljana, Slovenia

**Keywords:** axonal swelling, cationic pore-forming peptide, confocal laser scanning microscopy, equinatoxin II, ionic mechanisms, myelinated nerve fiber

## Abstract

This study was performed to elucidate the mechanism underpinning the nodal swelling induced by equinatoxin II (EqtII), a cation-selective pore-forming toxin derived from the sea anemone *Actinia equina*. Experiments were conducted using frog myelinated nerve fibers as a model system. Application of EqtII led to an approximately two-fold increase in the nodal volume of myelinated axons, but only when extracellular Ca^2+^ was present. Replacing extracellular Cl^−^ with isethionate had no measurable effect on this response, whereas substitution of NaCl with either sucrose or LiCl, an established Na^+^/Ca^2+^ exchanger (NCX) inhibitor, abolished the swelling. The persistence of the effect in the presence of tetrodotoxin indicates that voltage-gated Na^+^ channels are not involved in the underlying mechanism. Our data suggest that Ca^2+^ influx through EqtII-induced membrane pores raises intracellular Ca^2+^ levels, thereby stimulating the NCX in its forward-operating mode. This process promotes Ca^2+^ extrusion in exchange for Na^+^ entry. The resulting accumulation of intracellular Na^+^ increases osmotic pressure within the axon, leading to water influx and nodal swelling.

## 1. Introduction

Marine invertebrates represent a rich source of bioactive molecules with diverse ecological functions and potential pharmacological applications. Among them, sea anemones (phylum Cnidaria, class Anthozoa) produce a wide variety of peptide and protein toxins involved primarily in prey capture, predator deterrence and competition with other sessile organisms [1,2]. One of the best-characterized toxin families produced by sea anemones is the actinoporins. These biomolecules are soluble cytolytic proteins that bind to lipid membranes and oligomerize to form pores, thereby compromising membrane integrity and ultimately inducing cell lysis. Because of their well-defined mechanism of membrane permeabilization, these toxins have become important model systems for studying protein-lipid interactions and the molecular basis underlying pore formation [3,4,5,6]. Additionally, actinoporins have attracted interest for their potential applications in biotechnology and medicine, including targeted cytolytic agents and biosensing technologies [3,7,8,9,10].

**Figure 1:**
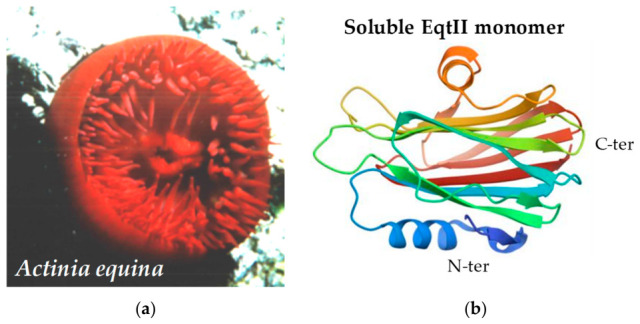
Sea anemone and structure of EqtII. (**a**) Photograph of the sea anemone *Actinia equina*, taken in the Mediterranean Sea. (**b**) Structure of the soluble EqtII monomer, based on PDB: 1IAZ, showing a compact β-sandwich fold and a mobile N-terminal helix (residues ~1–30) lying against the protein core in the soluble state.

A canonical representative of this family is equinatoxin II (EqtII) from the beadlet sea anemone *Actinia equina* (Figure 1). EqtII is a basic cytolytic protein of approximately 20 kDa composed of 179 amino acids and notably lacks cysteine residues and disulfide bonds, a structural feature conserved among actinoporins [11]. It belongs to a group of closely related toxins produced by *A. equina*, including EqtI and EqtIII, among which EqtII is the most abundant and best characterized isoform [12,13]. These proteins exhibit hemolytic and cytotoxic activities against a broad range of eukaryotic cells, primarily through membrane permeabilization. Early biochemical studies demonstrated that EqtII induces colloid-osmotic lysis of erythrocytes and other eukaryotic cells by forming membrane pores, leading to ionic imbalance and cell death [12,14,15]. Subsequent molecular and biophysical studies have revealed that the pore formation occurs through oligomerization of multiple toxin monomers (typically tetramers to octamers), with an estimated central pore of 1–2 nm in diameter, and have provided important insights into the lipid requirements of EqtII activity. Hence, membrane permeabilization by this toxin depends on the specific binding to sphingomyelin, a lipid abundant in eukaryotic plasma membranes, while membrane components such as cholesterol and lipid-raft-like domains can further enhance toxin binding and pore-forming efficiency [9,10,16,17,18].

Structurally, EqtII adopts a β-sandwich fold composed of twelve β-strands flanked by two short α-helices, forming a stable soluble monomer (Figure 1b). A highly flexible N-terminal segment (approximately residues 1–30) plays a critical role in membrane insertion and participates in the formation of the transmembrane pore [4,11,19]. Comparative analyses with other actinoporins, such as sticholysin-II from *Stichodactyla helianthus* and fragaceatoxin-C from *A. fragacea*, indicate that this N-terminal helix-mediated membrane insertion mechanism is conserved across members of the family [10,20].

Following EqtII-mediated pore formation, the uncontrolled flux of ions through these pores rapidly disrupts the electrochemical gradients that normally maintain cellular ionic homeostasis. In particular, the influx of mainly Ca^2+^ cations, combined with the efflux of intracellular solutes such as K^+^ cations, leads to a progressive osmotic imbalance across the plasma membrane. As water follows the ionic gradient, affected cells undergo swelling, a process that precedes membrane rupture and colloid-osmotic lysis [7,21,22,23].

Despite substantial progress in understanding the structural basis of EqtII pore formation and its lipid specificity, the cellular ionic mechanisms that drive the downstream process of toxin-induced cell swelling remain incompletely characterized. In particular, the relative contributions of different ionic fluxes and membrane transport pathways to the development of osmotic imbalance following pore formation are still not fully resolved. The objective of the present study was therefore to characterize in detail the ionic mechanisms and membrane pathways underlying EqtII-induced cell swelling using frog myelinated nerve fibers as a cellular model. By analyzing the effects of the toxin under controlled ionic conditions, we aimed to identify the ion fluxes responsible for osmotic imbalance and to clarify how EqtII-induced membrane permeabilization leads to the progressive swelling observed in intoxicated cells.

## 2. Results

### 2.1. EqtII-Induced Nodal Swelling

**Figure 2:**
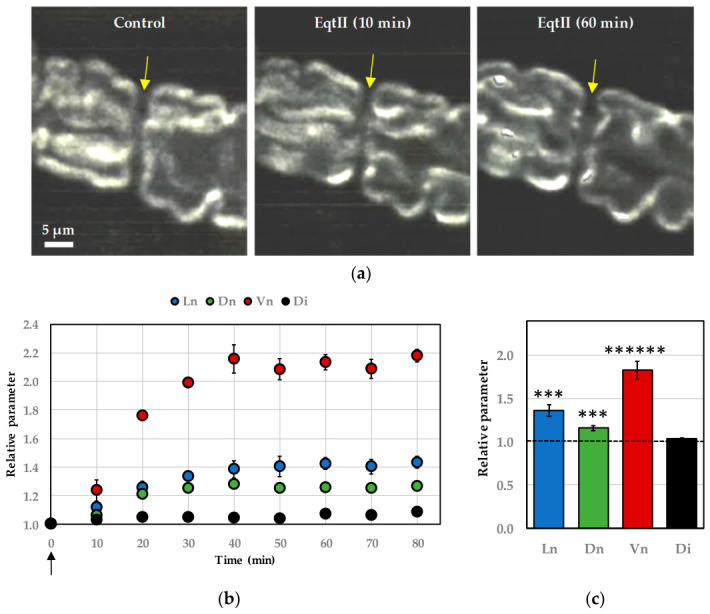
Effects of EqtII (10 nM) on the morphology of single myelinated nerve fibers bathed in Ringer’s solution. (**a**) Images of three-dimensional reconstructions by “look-through” projections calculated from series of optical sections of a myelinated axon (stained with FM1-43) before (**left**), and 10 min (**middle**) and 60 min (**right**) after EqtII addition to the extracellular medium. The arrows indicate the node of Ranvier. (**b**) Nodal length (Ln, blue circles), nodal diameter (Dn, green circles), nodal volume (Vn, red circles) and internodal diameter (Di, black circles) as a function of time, following EqtII addition at time zero (arrow) to the medium. The parameters are expressed relatively to those before toxin application. Mean ± S.E.M. calculated from five successive measurements of a representative myelinated axon. (**c**) Histogram of mean ± S.E.M. values, calculated from five successive measurements of each of fourteen myelinated axons, of nodal length (Ln, blue bar), nodal diameter (Dn, green bar), nodal volume (Vn, red bar) and internodal diameter (Di, black bar), after 60 min EqtII addition to the Ringer’s solution. The parameters are expressed relatively to those before toxin application (dashed line). ***: *p* < 0.0001 and ******: *p* < 0.0000001 (parametric paired two-tailed Student’s *t*-test).

The primary point questioned was to evaluate whether EqtII is capable of inducing nodal swelling under physiological conditions and to quantify the magnitude of this effect. To address this, myelinated nerve fibers were labeled with the fluorescent membrane probe N-(3-triethylammoniumpropyl)-4-(p-dibutylaminostyryl)pyridinium dibromide (FM1-43) and, after abundant washing of the probe with the Ringer’s solution, subsequently imaged using confocal laser scanning microscopy, enabling high-resolution visualization of the nodal architecture. As illustrated in Figure 2, application of 10 nM EqtII in Ringer’s solution led to distinct morphological changes localized at the nodes of Ranvier. These alterations were readily detectable by confocal imaging and indicate that EqtII exerts a measurable structural impact on nodal regions under near-physiological conditions.

Specifically, nodal length exhibited a clear and consistent increase, accompanied by a more modest expansion in nodal diameter. Together, these changes developed progressively, attained a plateau and resulted in an approximately two-fold increase in overall nodal volume (Figure 2b,c). In contrast, the diameter of the internodal regions remained stable throughout the observation period, indicating that the swelling effect was spatially restricted to the nodal domains rather than affecting the axon uniformly. This localized response suggests a targeted action of EqtII on nodal structures, potentially reflecting region-specific susceptibility or molecular interactions at the nodes of Ranvier.

### 2.2. Removal of Extracellular Ca^2+^ Prevents EqtII-Induced Nodal Swelling

**Figure 3:**
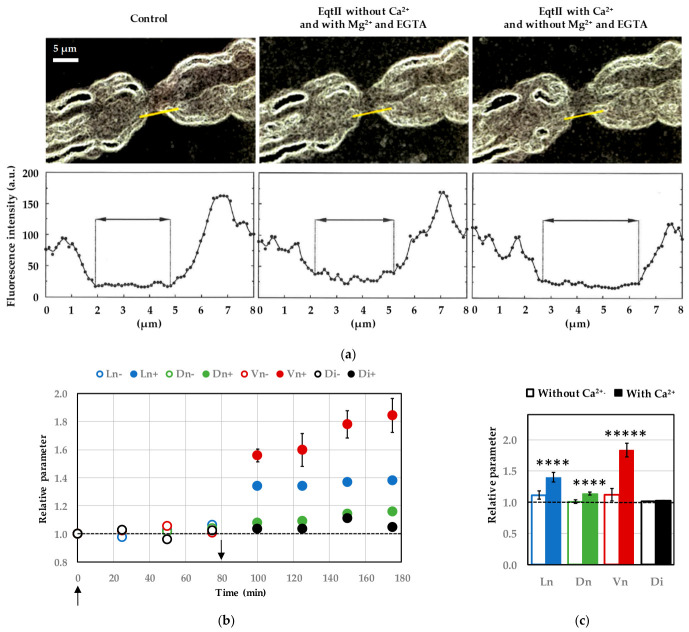
Effects of EqtII (10 nM) on the morphology of myelinated nerve fibers in the absence of extracellular Ca^2+^. (**a**) Images of three-dimensional reconstructions by “look-through” projections calculated from series of optical sections of a myelinated axon (stained with FM1-43, **upper**) and fluorescence intensity (**lower**) measured along the yellow line shown in the image above, in the Ringer’s solution (**left**), in the presence of EqtII added to a Ringer’s solution in which extracellular CaCl_2_ was substituted with MgCl_2_, combined with the addition of EGTA (**middle**) for 75 min (**middle**) and 95 min after EqtII addition to the Ringer’s solution (**right**). (**b**) Nodal length (Ln, blue circles), nodal diameter (Dn, green circles), nodal volume (Vn, red circles) and internodal diameter (Di, black circles) as a function of time, following EqtII addition at time zero (up arrow) to the Ringer’s solution in the absence (−, open circles) and presence (+, closed circles) of Ca^2+^ (indicated by the down arrow). The parameters are expressed relatively to those before toxin application (dashed line). Mean ± S.E.M. calculated from five successive measurements of a representative myelinated axon. (**c**) Histogram of mean ± S.E.M. values, calculated from five successive measurements of each of ten myelinated axons, of nodal length (Ln, blue bar), nodal diameter (Dn, green bar), nodal volume (Vn, red bar) and internodal diameter (Di, black bar), after EqtII addition to the Ringer’s solution devoid of Ca^2+^ for 75 min (open bars) and containing Ca^2+^ for 95 min (closed bars). The parameters are expressed relatively to those before toxin application (dashed line). ****: *p* < 0.00001 and *****: *p* < 0.000001 (parametric paired two-tailed Student’s *t*-test).

Substitution of extracellular CaCl_2_ with MgCl_2_, combined with the addition of ethylene glycol-bis(β-aminoethyl ether)-N,N,N′,N′-tetraacetic acid (EGTA) to chelate residual Ca^2+^, completely abolished the nodal swelling typically induced by EqtII (Figure 3). These conditions ensured a virtually Ca^2+^-free extracellular environment, thereby preventing Ca^2+^ influx. Accordingly, no significant alteration was observed in nodal length, nodal diameter, nodal volume or internodal diameter during 10 nM EqtII exposure (Figure 3b,c), indicating that the toxin alone is insufficient to induce morphological changes in the absence of extracellular Ca^2+^.

In contrast, when the Ringer’s solution containing CaCl_2_ was reintroduced after this initial period, EqtII triggered nodal swelling min (Figure 3). This was accompanied by significant increases in all nodal parameters, notably an approximately two-fold increase in nodal volume, with the exception of the internodal diameter that remained unchanged.

### 2.3. Extracellular Na^+^, but Not Cl^−^, Is Required for EqtII-Induced Nodal Swelling

**Figure 4:**
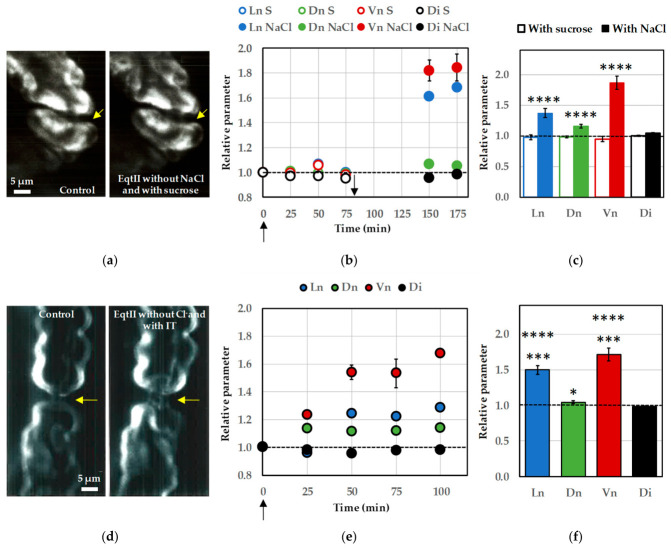
Effects of EqtII (10 nM) on the morphology of myelinated nerve fibers in the absence of extracellular Na^+^ or Cl^−^. (**a**) Images of a myelinated axon, in the Ringer’s solution (**left**) and in the presence of EqtII added to a Ringer’s solution in which extracellular NaCl was substituted isotonically with sucrose for 75 min (**right**). The arrows indicate the node of Ranvier. (**b**) Nodal length, nodal diameter, nodal volume and internodal diameter as a function of time, following EqtII addition at time zero (up arrow) to the medium in the absence (S, open circles) and presence (NaCl, closed circles) of NaCl (indicated by the down arrow). The parameters are expressed relatively to those before toxin application (dashed line). (**c**) Histogram of mean ± S.E.M. values, calculated from five successive measurements of each of twelve myelinated axons, of nodal length, nodal diameter, nodal volume and internodal diameter, after EqtII addition to the Ringer’s solution devoid of NaCl for 75 min (open bars) and containing NaCl for 95 min (closed bars). The parameters are expressed relatively to those before toxin application (dashed line). (**d**) Images of a myelinated axon before (**left**) and after (**right**) EqtII addition to the Ringer’s solution in which extracellular Cl^−^ was substituted with isethionate (IT) for 75 min. The arrows indicate the node of Ranvier. (**e**) Nodal length, nodal diameter, nodal volume and internodal diameter as a function of time, following EqtII addition at time zero (arrow) to the medium. The parameters are expressed relatively to those before toxin application (dashed line). (**f**) Histogram of mean ± S.E.M. values, calculated from five successive measurements of each of seventeen myelinated axons, of nodal length, nodal diameter, nodal volume and internodal diameter, after 100 min EqtII addition to the Ringer’s solution in which extracellular Cl^−^ was substituted with isethionate. The parameters are expressed relatively to those before toxin application (dashed line). See Figure 2 and Figure 3 legends for more details. *: *p* < 0.05, ****: *p* < 0.00001 and *******: *p* < 0.00000001 (parametric paired two-tailed Student’s *t*-test).

Replacement of extracellular NaCl with sucrose completely suppressed the morphological effects normally elicited by EqtII (Figure 4a–c), demonstrating that the presence of extracellular Na^+^ and/or Cl^−^ is a key determinant of toxin-induced structural changes.

Under these NaCl-free conditions, nodal length and nodal diameter remained comparable to baseline values and, consequently, no increase in nodal volume was observed during EqtII exposure (Figure 4b,c). This complete absence of swelling strongly supports the conclusion that ion-dependent osmotic processes, most likely involving Na^+^ and/or Cl^−^, underly the expansion of nodal structures induced by the toxin. In contrast, when the Ringer’s solution containing NaCl was reintroduced after this initial period, EqtII triggered nodal swelling within 95 min. This was accompanied by significant increases in all nodal parameters, notably an approximately two-fold increase in nodal volume (Figure 4b,c).

However, when extracellular Cl^−^ was replaced with isethionate, EqtII retained its full capacity to induce nodal swelling (Figure 4d–f). Under these conditions, nodal length increased markedly, accompanied by a moderate but consistent enlargement of nodal diameter (Figure 4e,f). As a result, nodal volume still exhibited a substantial rise, similar to that observed under control conditions (see Figure 2). These findings indicate that Cl^−^ ions are not required for the structural remodeling triggered by EqtII.

Importantly, under both ionic conditions, the internodal diameter remained unchanged throughout the experiments (Figure 4b,c,e,f). This stability confirms that the observed effects are highly localized to the nodes of Ranvier and do not reflect a generalized swelling of the entire axon.

Altogether, these results highlight a selective dependence on extracellular Na^+^ for EqtII-induced increases in nodal length, diameter and volume, and reinforce the spatial specificity of the toxin action at nodal regions.

### 2.4. Voltage-Gated Na^+^ Channels Are Not Required for EqtII-Induced Nodal Swelling

**Figure 5:**
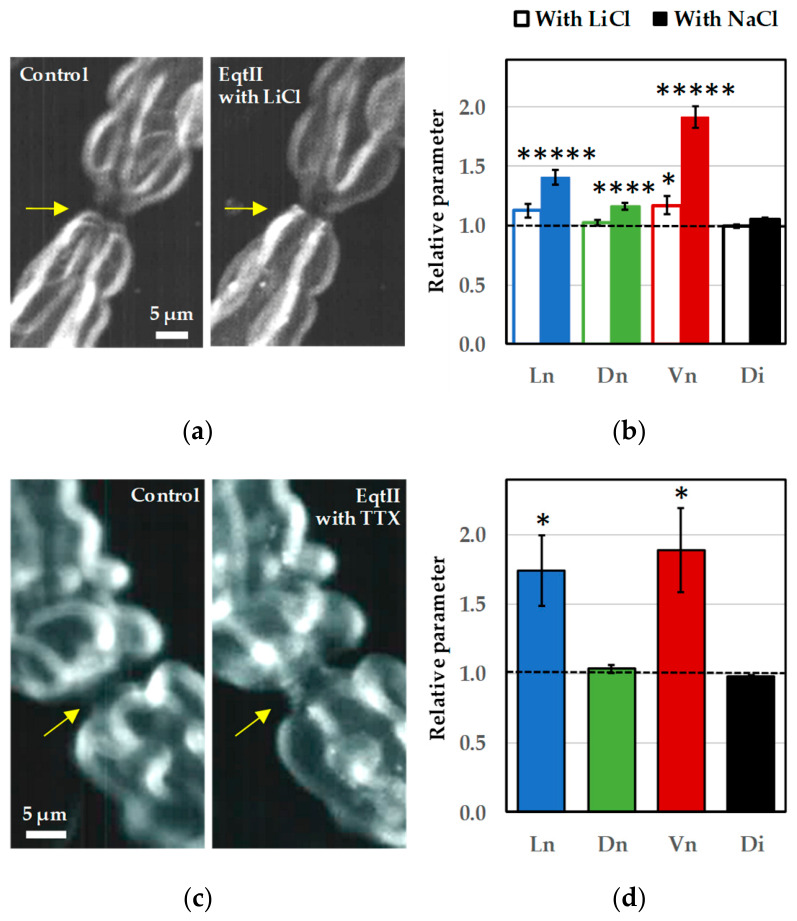
Effects of EqtII (10 nM) on the morphology of myelinated nerve fibers in a Ringer’s solution in which extracellular Na^+^ was substituted with Li^+^ or added with TTX (1 µM). (**a**) Images of a myelinated axon, in the Ringer’s solution (**left**) and in the presence of EqtII added to a Ringer’s solution in which extracellular NaCl was substituted with LiCl for 100 min (**right**). The arrows indicate the node of Ranvier. (**b**) Histogram of mean ± S.E.M. values, calculated from five successive measurements of each of twelve myelinated axons, of nodal length, nodal diameter, nodal volume and internodal diameter, after EqtII addition to the medium containing LiCl for 100 min (open bars) and then NaCl for 90 min (closed bars). The parameters are expressed relatively to those before toxin application (dashed line). (**c**) Images of a myelinated axon before (**left**) and after (**right**) EqtII and TTX addition to the Ringer’s solution for 95 min. The arrows indicate the node of Ranvier. (**d**) Histogram of mean ± S.E.M. values, calculated from five successive measurements of each of twelve myelinated axons, of nodal length, nodal diameter, nodal volume and internodal diameter, after 95 min EqtII and TTX addition to the Ringer’s solution. The parameters are expressed relatively to those before toxin application (dashed line). See Figure 2 and Figure 3 legends for more details. *: *p* < 0.05, ****: *p* < 0.00001 and *****: *p* < 0.000001 (parametric paired two-tailed Student’s *t*-test).

To determine whether voltage-gated Na^+^ (Na_V_) channels contribute to the extracellular Na^+^ requirement for EqtII-induced nodal swelling, NaCl in the bathing solution was replaced with LiCl, as these channels are reported to exhibit similar selectivity for Na^+^ and Li^+^ [24]. Under these conditions, exposure to EqtII failed to induce any detectable structural changes at the nodes of Ranvier (Figure 5a,b). Nodal length and diameter remained at baseline levels, and no increase in nodal volume was observed during toxin exposure.

Reintroduction of NaCl in the continued presence of the toxin restored the swelling response (Figure 5b). This response was characterized by a marked elongation of the nodal region accompanied by a moderate increase in nodal diameter, resulting in an approximately two-fold increase in nodal volume. In contrast, internodal diameter remained stable throughout the time, indicating, once again, that the effect was spatially restricted to the nodes of Ranvier.

These findings demonstrate that extracellular Na^+^ is essential for the development of EqtII-induced nodal swelling and cannot be functionally replaced by Li^+^. This suggests the involvement of Na^+^-specific entry pathways other than Na_V_ channels.

To further assess whether Na_V_ channels contribute to EqtII-induced nodal swelling, myelinated nerve fibers were treated with tetrodotoxin (TTX, 1 µM), a selective blocker of these channels [25,26]. Consistent with the above results, TTX did not prevent the morphological effects of EqtII (Figure 5c,d). Hence, in the presence of TTX, exposure to EqtII still produced a pronounced increase in nodal length, accompanied by a moderate enlargement of nodal diameter. Consequently, nodal volume increased substantially, reaching values comparable to those observed in the absence of TTX (Figure 5d). As under all other experimental conditions, the internodal diameter remained unchanged (Figure 5d), confirming that the swelling response was highly localized to the nodes of Ranvier and did not reflect generalized axonal expansion.

Taken together, these findings demonstrate that EqtII-induced nodal swelling does not require Na_V_ channel activity. When considered together with the strict dependence on extracellular Na^+^, this result suggests that Na^+^ entry occurs through a TTX-insensitive pathway, potentially involving alternative Na^+^-permeable routes that bypass classical Na_V_ channels.

## 3. Discussion

The present study provides new insight into the ionic mechanisms underlying the nodal swelling induced by EqtII, a canonical actinoporin, in myelinated axons. Using frog myelinated nerve fibers and high-resolution confocal imaging, we demonstrate that EqtII triggers a pronounced swelling selectively localized at the nodes of Ranvier. This response is characterized by a marked increase in nodal length, accompanied by a more moderate expansion in nodal diameter, resulting in an approximately two-fold increase in nodal volume, while internodal regions remain unaffected.

### 3.1. Localized Nodal Vulnerability to EqtII

EqtII does not induce a generalized osmotic swelling of the axon, but rather a highly localized structural remodeling of the nodal domains. The spatial restriction of swelling to the nodes of Ranvier highlights their intrinsic vulnerability. Nodes of Ranvier are highly specialized membrane domains enriched in ion channels and exchangers characterized by the absence of compact myelin [27], which likely facilitates EqtII access and membrane insertion. In addition, the sphingomyelin-rich composition of nodal membranes strongly favors EqtII binding and oligomeric pore formation [5,6,16,17,18]. This lipid dependence may contribute to the selective vulnerability of nodal regions. Conversely, the lack of detectable changes in internodal diameter suggests that compact myelin acts as a physical and functional barrier limiting toxin access and buffering osmotic stress. Together, these observations explain the selective nodal vulnerability to pore-forming toxins and reinforce the biological relevance of the model used.

### 3.2. Essential Role of Extracellular Ca^2+^

Our experiments demonstrate the absolute requirement for extracellular Ca^2+^ in the development of EqtII-induced nodal swelling. Under Ca^2+^-free conditions, the toxin failed to produce any measurable changes in nodal morphology, despite its ability to interact with membranes. The reintroduction of Ca^2+^ restored the swelling nodal response, demonstrating that Ca^2+^ influx is a critical initiating event to subsequent ionic rearrangements. This observation is consistent with previous studies showing that EqtII forms cation-selective pores permeable to Ca^2+^ and induces an increase in intracellular Ca^2+^ concentration [7,21,22,23]. In particular, microspectrofluorometric studies using NG108-15 neuroblastoma cells preloaded with the Ca^2+^ indicator fura-2/AM showed that EqtII induces a marked elevation of intracellular Ca^2+^ concentration [22]. Such a rise was attributed to passive Ca^2+^ influx through toxin-induced pores in the plasma membrane, rather than to the activation of voltage-gated Ca^2+^ channels.

Beyond its direct contribution to ionic flux, Ca^2+^ is a well-known key regulator of multiple intracellular processes and membrane transport systems. Its entry into the axon is therefore likely to act as both a trigger and an amplifier of downstream events leading to osmotic imbalance.

### 3.3. Extracellular Na^+^, but Not Cl^−^, Is Strictly Required

Our results also demonstrate that extracellular Na^+^ is strictly required for nodal swelling, whereas Cl^−^ ions are not involved. Hence, replacement of NaCl with sucrose or LiCl abolished or significantly reduced the nodal swelling, while substitution of Cl^−^ with isethionate had no effect. These findings clearly establish that Na^+^ influx, rather than generalized ionic imbalance or Cl^−^ movement, is the primary driver of the osmotic changes leading to nodal swelling.

The inability of Li^+^ to substitute for Na^+^ is particularly informative. Although Li^+^ can permeate certain cationic pathways such as Na_V_ channels, it is not efficiently transported by Na^+^-dependent exchangers such as the Na^+^/Ca^2+^ exchanger (NCX), and may even interfere with its function [28]. This observation strongly suggests that Na^+^ entry occurs through specific, regulated transport mechanisms rather than solely through Na_V_ channels or through passive diffusion via toxin-induced pores.

### 3.4. TTX-Insensitivity Excludes Voltage-Gated Sodium Channels

This interpretation is further supported by the observation that TTX did not inhibit EqtII-induced nodal swelling. Despite the high density of Na_V_ channels at the nodes of Ranvier, their blockade by TTX, a potent and highly specific inhibitor of these channels [25,26], had no detectable effect on the extent of EqtII-induced swelling. The persistence of swelling in the presence of TTX clearly indicates that Na_V_ channels are neither necessary nor sufficient for the Na^+^ influx required for driving nodal swelling.

This finding, combined with the strict dependence on extracellular Na^+^, confirms that Na^+^ entry occurs through TTX-insensitive pathways such as secondary activation of Na^+^-dependent transport systems.

### 3.5. K^+^ Movements Do Not Represent the Dominant Mechanism Underlying Nodal Swelling

An additional point that merits consideration is the potential contribution of K^+^ movements to the ionic disturbances induced by EqtII. Actinoporin pores, including those formed by EqtII, are known to be permeable to monovalent cations, such as K^+^ [5,16]. Consequently, toxin-induced K^+^ efflux may occur following pore formation and could theoretically contribute to alterations in intracellular ionic homeostasis. However, from a biophysical point of view, the loss of intracellular K^+^ would not be expected to generate the osmotic driving force required for water influx and nodal swelling. In addition, the response is strictly dependent on extracellular Na^+^ and Ca^2+^ and persists under conditions where Na^+^ is selectively manipulated, indicating that inward cation fluxes are the major determinants of the osmotic response. Therefore, while K^+^ movements may nonetheless participate in secondary ionic redistribution following pore formation, they are unlikely to represent the primary mechanism responsible for EqtII-induced nodal swelling.

### 3.6. Proposed Mechanism: Coupling Between Ca^2+^ Influx and Na^+^ Entry via NCX

Taken together, our results support a mechanistic model in which EqtII-induced swelling arises from a functional coupling between Ca^2+^ influx and Na^+^ accumulation. Following membrane insertion, EqtII forms pores that allow Ca^2+^ entry into the axon. The resulting increase in intracellular Ca^2+^ is expected to activate the NCX operating in its forward mode, whereby Ca^2+^ is extruded in exchange for Na^+^ entry, leading to Na^+^ accumulation and thus to osmotic water influx and nodal swelling [28].

**Figure 6:**
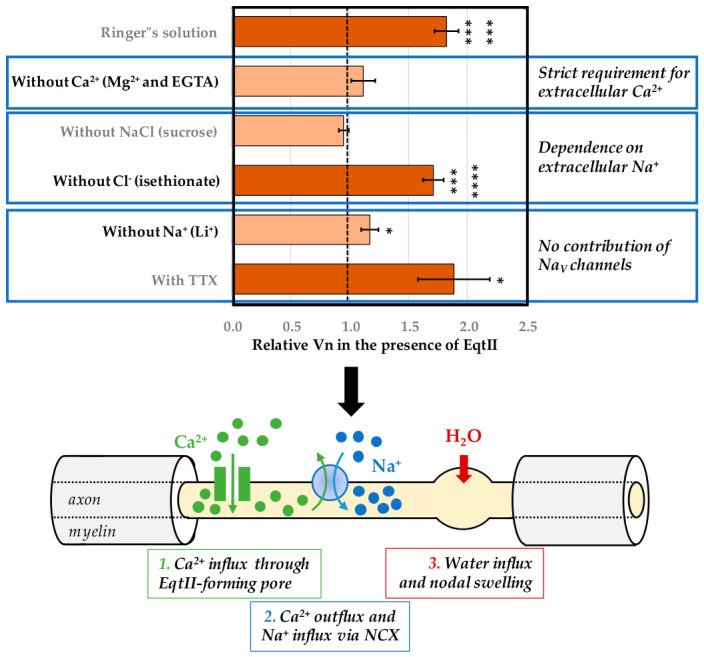
Ionic determinants and proposed mechanism of EqtII-induced nodal swelling of myelinated axons. (**Top**) Histogram of relative mean ± S.E.M. values of nodal volume (Vn) following EqtII (10 nM) application under the indicated different conditions, and normalized to control (dashed line). See Figure 2, Figure 3, Figure 4 and Figure 5 legends for more details. *: *p* < 0.05, ******: *p* < 0.0000001 and *******: *p* < 0.00000001. (**Bottom**) Schematic representation of the proposed mechanism underlying EqtII-induced nodal swelling. (1) EqtII forms pores in the nodal membrane, allowing Ca^2+^ influx into the axon. (2) The resulting increase in intracellular Ca^2+^ activates the NCX, which extrudes Ca^2+^ in exchange for Na^+^ entry. (3) Intracellular Na^+^ accumulation generates an osmotic gradient that drives water influx, leading to localized swelling at the node of Ranvier (yellow area). Internodal regions (grey areas) remain unaffected due to the insulating properties of the compact myelin.

This mechanistic model accounts for the following key observations (Figure 6): (i) the strict requirement for extracellular Ca^2+^, (ii) the dependence on extracellular Na^+^, (iii) the inability of Li^+^ to substitute for Na^+^, and (iv) the lack of effect of TTX. Through NCX activity, Ca^2+^ influx is effectively converted into intracellular Na^+^ accumulation, leading to an increase in osmotic pressure. Water entry then follows, resulting in the observed swelling of nodal regions. The EqtII-induced influx of Ca^2+^ and the secondary Na^+^ entry via NCX illustrate how even non-specific pores can perturb highly localized ionic domains, producing morphological changes such as nodal swelling of myelinated nerve fibers.

Although direct Na^+^ permeation through EqtII pores may contribute minimally to this process, the ionic selectivity of the response strongly supports a predominant role for transporter-mediated Na^+^ influx. This mechanistic interpretation is fully consistent with previous reports linking pore-forming toxins, Ca^2+^ influx, NCX activity and osmotic swelling [21,22,23,28,29].

### 3.7. Physiological Relevance and Pathophysiological Implications

The present findings have important implications for understanding how pore-forming toxins disrupt neuronal structure and function. By selectively targeting nodal regions of myelinated nerve fibers and exploiting endogenous ion transport systems, EqtII induces localized swelling that is likely to impair saltatory conduction. In particular, nodal swelling is expected to reduce nerve conduction velocity by altering nodal geometry and increasing nodal membrane capacitance. An early pioneering study reported functional alterations in isolated frog single myelinated fibers exposed to the toxin, including increased membrane permeability and altered excitability, thereby establishing the first link between equinatoxin exposure and disruption of myelinated nerve fiber physiology [30]. Structural changes in nodal geometry may further disrupt the precise organization and distribution of ion channels, thereby compromising action potential propagation, and potentially leading to severe consequences for neuronal signaling. In addition, recent studies show that NCX can transiently reverse or operate in mixed modes under pathological conditions, including ischemia and excitotoxicity [31,32].

More broadly, this study extends our understanding of how actinoporins disrupt excitable membranes and highlights the importance of secondary ionic mechanisms in toxin-induced cellular injury. Rather than acting solely through passive membrane permeabilization, EqtII appears to amplify its effects by engaging endogenous transport systems that exacerbate ionic imbalance and osmotic stress.

### 3.8. Limitations and Future Directions

The reduction in nodal swelling observed upon Na^+^ substitution with Li^+^ should be interpreted with caution since, in neurons and myelinated axons, Li^+^ has been shown to affect Na^+^-dependent processes and ionic homeostasis, particularly under conditions of stress or altered ion gradients, where Na^+^ and Ca^2+^ dysregulation play a central role in axonal physiology and pathology [33]. In addition, Li^+^ can influence intracellular signaling pathways, including phosphoinositide metabolism through the inhibition of inositol (1,4,5)-trisphosphate signaling, which may affect ion transport and volume regulation [34]. However, how this mechanism could influence nodal swelling remains unclear. More broadly, the intracellular signaling cascades regulating nodal volume are still poorly understood, highlighting important directions for future investigation.

Although the proposed involvement of NCX provides a coherent mechanistic framework, it is important to emphasize that this involvement is inferred indirectly. While the dependence on Ca^2+^ and Na^+^, the failure of Li^+^ substitution and TTX insensitivity strongly support this hypothesis, direct experimental evidence remains to be obtained. In particular, future studies using specific NCX inhibitors or direct measurements of intracellular Na^+^ and Ca^2+^ dynamics during EqtII exposure would provide definitive confirmation of this mechanistic framework. Therefore, NCX involvement should be considered as a well-supported working hypothesis, which requires further experimental validation using complementary approaches.

It would also be of interest to examine whether similar mechanisms operate in other cell types and with other actinoporins, to generalize our findings, as well as to explore the potential modulation of these effects by membrane lipid composition. Additionally, comparative approaches may clarify the minimal structural determinants required for Ca^2+^ permeability versus mere lytic activity.

## 4. Materials and Methods

### 4.1. Toxins and Chemicals

Equinatoxin II (Figure 1b) was generously supplied by Dr. P. Macek (University of Ljubljana, Slovenia). The methods for toxin isolation and purification have been described previously [13]. EqtII was dissolved in distilled water containing 1% bovine serum albumin (BSA) and aliquoted into multiple portions. TTX citrate (molecular weight 319.27, purity > 98%) was obtained from Sigma-Aldrich (St. Quentin-Fallavier, France), and was prepared in phosphate-buffered saline (PBS, 1×) supplemented with 10% phosphate buffer to yield a 2.85-mM stock solution. The stock solutions were stored at −20 °C and diluted immediately prior to use in the appropriate experimental media to obtain the final toxin concentrations specified in the text.

Sodium isethionate was sourced from Matheson Coleman and Bell (East Rutherford, NJ, USA) while other chemicals were purchased from Sigma-Aldrich (St. Quentin-Fallavier, France). The fluorescent probe FM1-43 was acquired from Molecular Probes Europe (Leiden, The Netherlands). All salts employed in the experiments were of analytical grade.

### 4.2. Animals and Myelinated Nerve Fibers

Experiments were conducted on single myelinated axons composing the sciatic nerves isolated from adult male frogs (*Rana esculenta*, weighing 20–25 g). The animals were bred and maintained in a natural pond at the CNRS campus in Gif-sur-Yvette, France. They were euthanized by double pithing immediately prior to use for experiments during the September–October period. All efforts were made to reduce both the number of animals used and any associated discomfort.

Individual myelinated axons were carefully separated from a desheathed segment of sciatic nerve (~2 cm in length) and pinned to the bottom of a 2-mL Plexiglas recording chamber lined with Rhodorsil^®^ (Sigma-Aldrich, St. Quentin-Fallavier, France), following previously described procedures [35]. The axons were incubated with FM1-43 (2 µM) in the dark for 15 min at 21–22 °C to allow dye incorporation into lipids, after which excess dye was removed by washing with dye-free solution before imaging. This procedure stained the various membrane structures of myelinated axons for at least 120 min without destaining (no detectable photobleaching or redistribution).

The standard physiological Ringer’s solution contained (in mM): NaCl 115, KCl 2, CaCl_2_ 1.8 and HEPES (4-(2-hydroxyethyl)-1-piperazineethanesulfonic acid) 5 (pH adjusted to 7.4 with NaOH, osmolality of 244.4 mOsm). In certain experiments, a nominally Ca^2+^-free solution was prepared by replacing CaCl_2_ (1.8 mM) with MgCl_2_ (1.8 mM) and adding EGTA (2 mM). In other experiments, NaCl (115 mM) was substituted with either sucrose (230 mM), sodium isethionate (115 mM) or LiCl (115 mM), while maintaining constant osmolality. The osmolality of solutions was systematically controlled using a freezing-point osmometer (Knauer, Berlin, Germany) to ensure similar osmolality (±5.0 mOsm) and thus avoiding changes in osmotic balance.

### 4.3. Confocal Laser Scanning Microscopy

Myelinated nerve fibers were imaged with a Sarastro 2000 confocal laser scanning microscope (Molecular Dynamics, Sunnyvale, CA, USA), composed of an upright microscope (Nikon Optiphot-2, Nikon Europe B.V., Amstelveen, The Netherlands) equipped with a single argon laser. The confocal pinhole aperture was set to 100 µm. Imaging was performed using a 40× water-immersion objective (Zeiss, Rueil-Malmaison, France; numerical aperture 0.75). Excitation was achieved with the 488 nm line of the Argon ion laser, and fluorescence emission was collected through a 510 nm long-pass filter. Series of optical sections were acquired at intervals of 0.5–0.7 µm using a standard format of 512 × 512 pixels. Three-dimensional reconstructions were subsequently generated using “look-through” projection methods, as described previously [35]. All experiments were carried out at controlled room temperature (21–22 °C).

**Figure 7:**
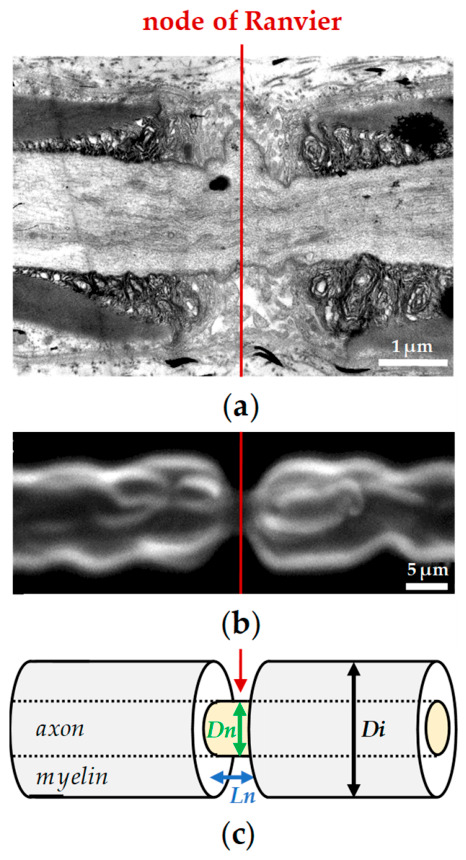
The myelinated axon of frog sciatic nerve. (**a**) Electron micrograph of the nodal region (Credit: Leonardo Mateu). Note the clear distinction between central nodal gap, neighboring paranodes and myelinated regions. (**b**) Confocal laser scanning microscopy of a myelinated axon stained with FM1-43. The image is a three-dimensional reconstruction by a “look-through” projection calculated from series of optical sections scanned at 0.5 µm increments. (**c**) Schematic representation of a myelinated nerve fiber. The simplest geometry of nodal (in yellow) and internodal (in grey) parts of the fiber is assumed to correspond to co-axial cylinders. Ln and Dn are nodal length and diameter, respectively, and Di is internodal diameter. In each case, the red arrow indicates the node of Ranvier.

Quantitative analyses were performed on the same myelinated axon before and during the different experimental conditions. For each fiber, nodal volume (Vn) was estimated using the relation Vn = πLn(Dn/2)^2^, where Ln and Dn correspond to the nodal length and diameter, respectively, measured on three-dimensional projections, assuming a cylindrical geometry for the node of Ranvier (Figure 7).

### 4.4. Statistical Analysis

Each parameter value is presented as the mean ± S.E.M., calculated from five successive measurements of each *n* independent experiments. Statistical comparisons were carried out using the parametric paired two-tailed Student’s *t*-test, with differences considered statistically significant at *p* < 0.05 (*: *p* < 0.05, **: *p* < 0.001, ***: *p* < 0.0001, ****: *p* < 0.00001, *****: *p* < 0.000001, ******: *p* < 0.0000001 and *******: *p* < 0.00000001).

## 5. Conclusions

In conclusion, our study demonstrates that EqtII, a cationic pore-forming actinoporin, induces selective swelling at the nodes of Ranvier of myelinated axons. This swelling is characterized by marked increases in nodal length and moderate enlargement of nodal diameter, resulting in a substantial rise in nodal volume, while internodal regions remain unaffected. We establish that EqtII-induced swelling requires extracellular Ca^2+^ and Na^+^, is independent of Cl^−^ and occurs in the absence of Na_V_ channel activity. These findings support a mechanism in which Ca^2+^ influx through EqtII pores triggers Na^+^ accumulation, likely via the forward mode of NCX, generating localized osmotic stress that drives water entry and nodal swelling. The inability of Li^+^ to substitute for Na^+^ further reinforces the potential central role of NCX in mediating this process. However, while this mechanism is strongly supported by indirect experimental evidence, it should be considered as a working hypothesis rather than a definitive conclusion. Therefore, further studies are required to directly confirm the involvement of NCX and to assess the contribution of additional ionic pathways.

Collectively, these results reveal a novel, transporter-mediated amplification mechanism by which pore-forming toxins exploit endogenous ionic pathways to induce highly localized structural remodeling of excitable membranes. Beyond its relevance to actinoporin biology, this work provides a mechanistic framework for understanding how membrane-permeabilizing toxins compromise axonal integrity and may inform future therapeutic strategies aimed at modulating Na^+^/Ca^2+^-dependent osmotic stress in neurons.

## Data Availability

All data are included in the present work and are available from the corresponding author upon reasonable request.

## Abbreviations

The following abbreviations are used in this manuscript:

BSA	Bovine serum albumin
Di	Internodal diameter
Dn	Nodal diameter
EGTA	Ethylene glycol-bis(β-aminoethyl ether)-N,N,N′,N′-tetraacetic acid
EqtII	Equinatoxin-II
FM1-43	N-(3-triethylammoniumpropyl)-4-(p-dibutylaminostyryl)pyridinium dibromide
HEPES	4-(2-Hydroxyethyl)-1-piperazineethanesulfonic acid
Ln	Nodal length
Na_V_ channels	Voltage-gated Na^+^ channels
NCX	Na^+^/Ca^2+^ exchanger
PBS	Phosphate-buffered saline
TTX	Tetrodotoxin
Vn	Nodal volume
